# Angioplasty is an Effective Treatment for Vasospasm Following Pituitary Apoplexy and Tumor Resection

**DOI:** 10.7759/cureus.2117

**Published:** 2018-01-26

**Authors:** Diana G Douleh, Peter J Morone, Bret Mobley, Matthew R Fusco, Lola B Chambless

**Affiliations:** 1 Department of Orthopedic Surgery, University of Colorado; 2 Department of Neurological Surgery, Vanderbilt University Medical Center; 3 Department of Pathology, Microbiology and Immunology, Vanderbilt University Medical Center; 4 Vanderbilt University Medical Center

**Keywords:** pituitary apoplexy, vasospasm, endovascular, angioplasty, verapamil

## Abstract

Pituitary apoplexy is a clinical syndrome characterized by acute headache, visual changes, and decreased consciousness occurring in association with hemorrhage or infarct of an existing pituitary adenoma. Surgical management involves tumor resection and decompression of surrounding structures including the optic apparatus. Vasospasm is a rare but potentially devastating complication of pituitary apoplexy. We present a case of pituitary apoplexy in a 28-year-old male treated with emergent endoscopic transsphenoidal resection. On postoperative day seven, following  surgical resection, the patient developed neurologic deficits and motor weakness, and severe vasospasm was diagnosed. This is a novel case of intra-arterial verapamil and angioplasty used to treat vasospasm following surgical decompression for pituitary apoplexy. The patient experienced complete recovery of motor deficits following treatment. The authors propose angioplasty as an effective treatment for postoperative vasospasm following transphenoidal surgery for pituitary apoplexy in the presence of focal vessel stenosis.

## Introduction

Pituitary apoplexy is a clinical syndrome associated with hemorrhage or infarct of an existing pituitary adenoma characterized by some combination of acute severe headache, visual impairment, ophthalmoplegia, pituitary hormonal dysfunction, and altered mental status [1-2]. Management involves high-dose corticosteroids and, in patients with acute vision loss or cranial neuropathy, surgical decompression [1-2]. Vasospasm is a rare complication of pituitary apoplexy. We present a novel case of post-apoplexy vasospasm successfully treated with intra-arterial verapamil and angioplasty.

## Case presentation

History and examination

**Figure 1:**
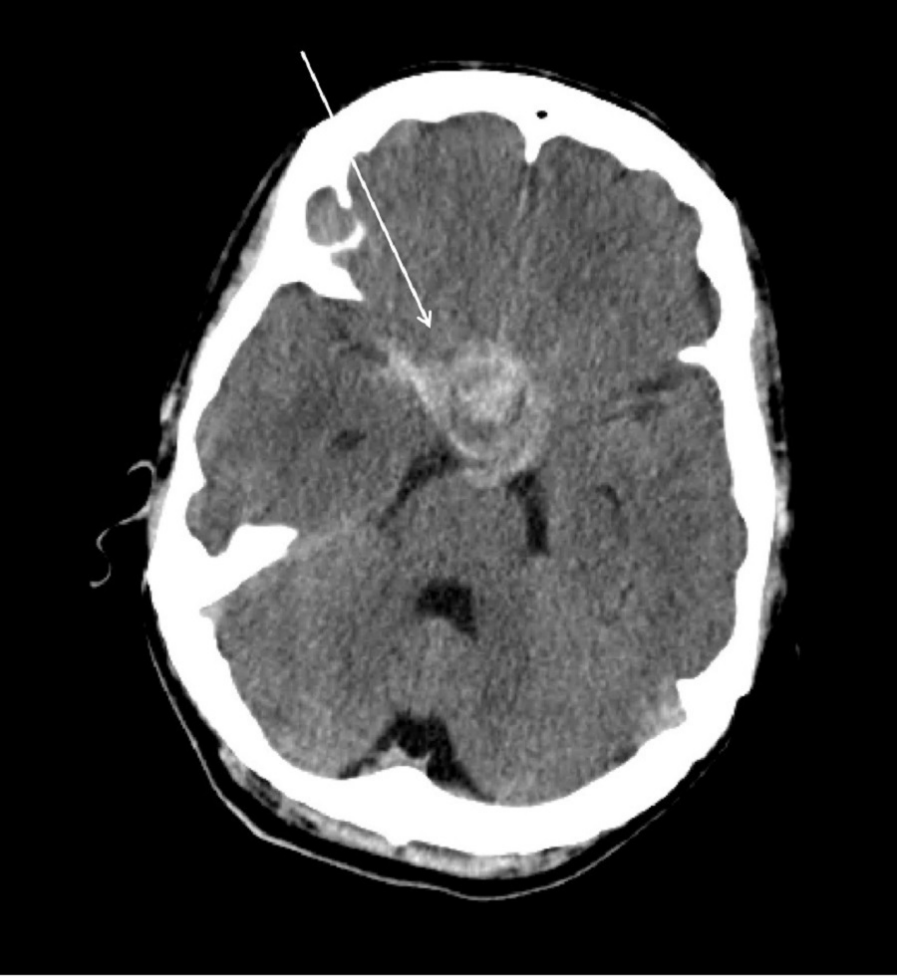
Preoperative head computed tomography (CT) without contrast demonstrating sellar mass extending into the left cavernous sinus.

The patient, a 28-year-old male with a history of migraines, presented to an emergency department complaining of acute visual change, frontal headache, nausea, and vomiting with worsening symptoms over two weeks duration. On physical examination, the patient was completely blind with no light perception in both eyes; his pupils were 4 mm and sluggishly reactive to light, and a left cranial nerve (CN) VI palsy was noted. The patient was otherwise neurologically intact. Magnetic resonance imaging (MRI) demonstrated a heterogeneously enhancing 4 x 3.2 x 2.2 cm sellar mass with 4 cm extension into the adjacent suprasellar compartment. The mass demonstrated compression and elevation of the optic chiasm, and extensive leptomeningeal enhancement. The patient was subsequently transferred to our institution. At the time of our evaluation, the patient continued to have poor vision with persistent absence of bilateral light perception and a left CN VI palsy. Computed tomography (CT) at our institution showed a large sellar and suprasellar mass measuring 4 cm x 4 cm, displacing the third ventricle, extending into the left cavernous sinus, and eroding into the superior aspect of the clivus (Figure 1). Laboratory studies revealed a serum sodium of 157 millimoles/liter and urine specific gravity of 1.002. Urine output was found to be 550 milliliters (mL) per hour consistent with diabetes insipidus. Hydrocortisone 50 mg and desmopressin 1 mg were administered. Of note, the patient’s prolactin level was low at 1.0 nanograms/mL.

Operative course

**Figure 2:**
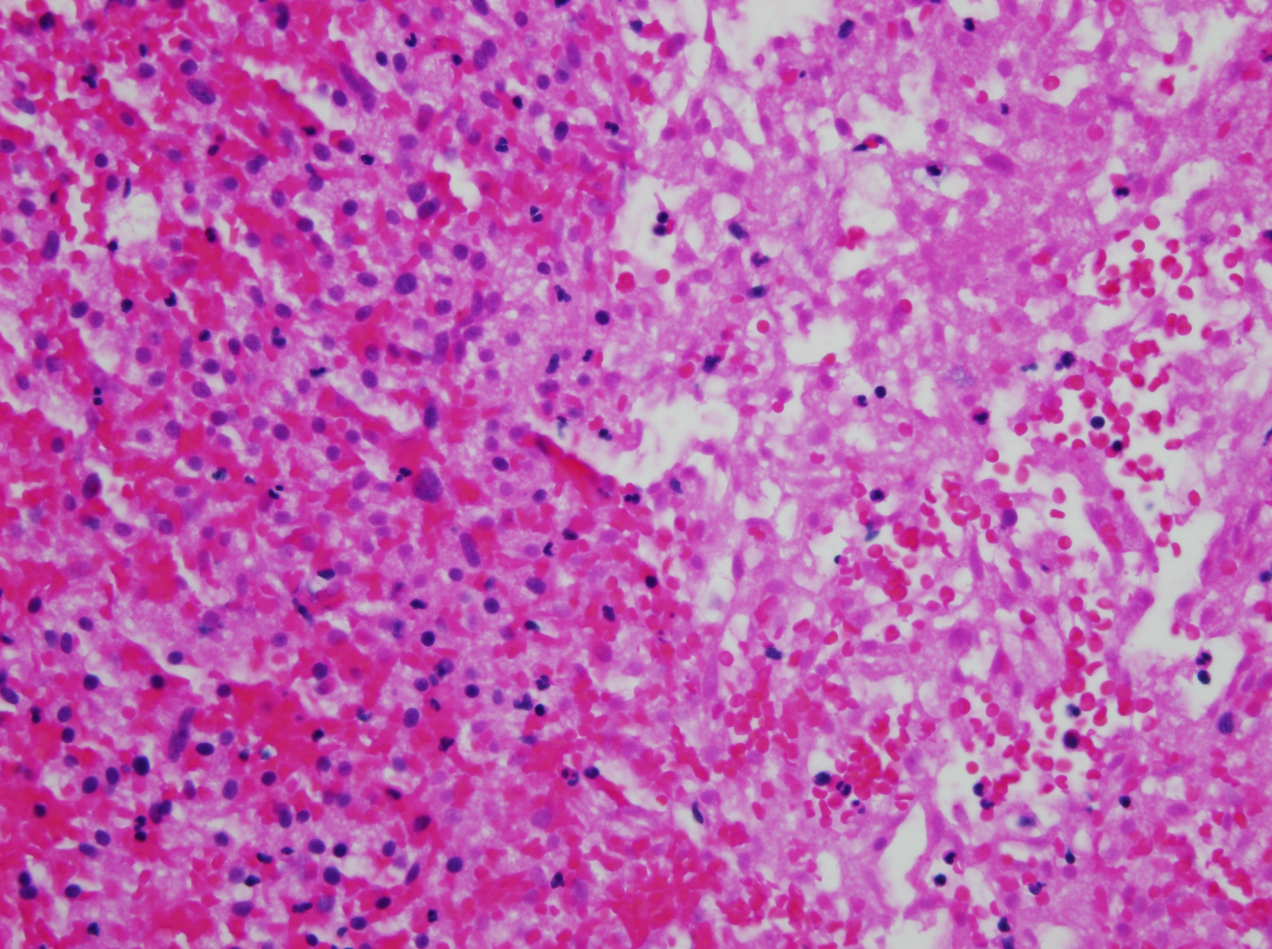
Widespread cytoplasmic eosinophilia was present in the tumor cells, indicative of ischemic change, and areas of frank necrosis and acute hemorrhage were present.

**Figure 3:**
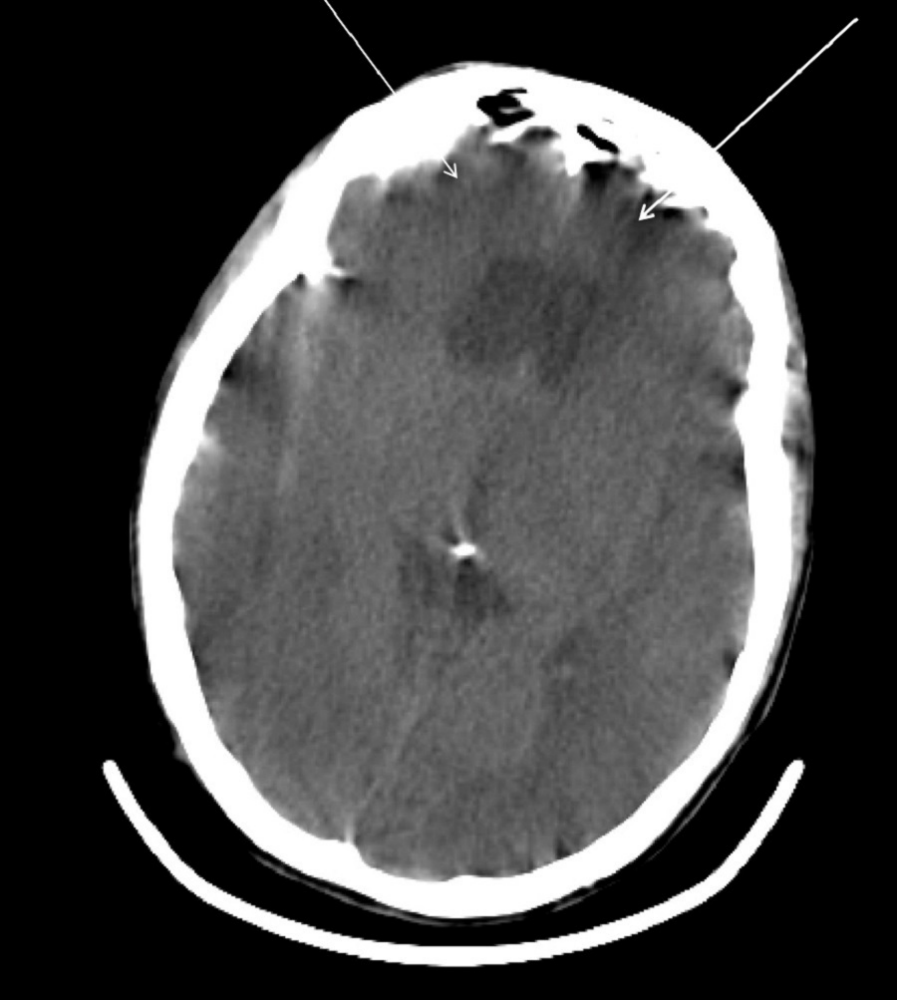
Postoperative head computed tomography (CT) without contrast demonstrating new acute infarcts involving the bilateral frontal lobes.

**Figure 4:**
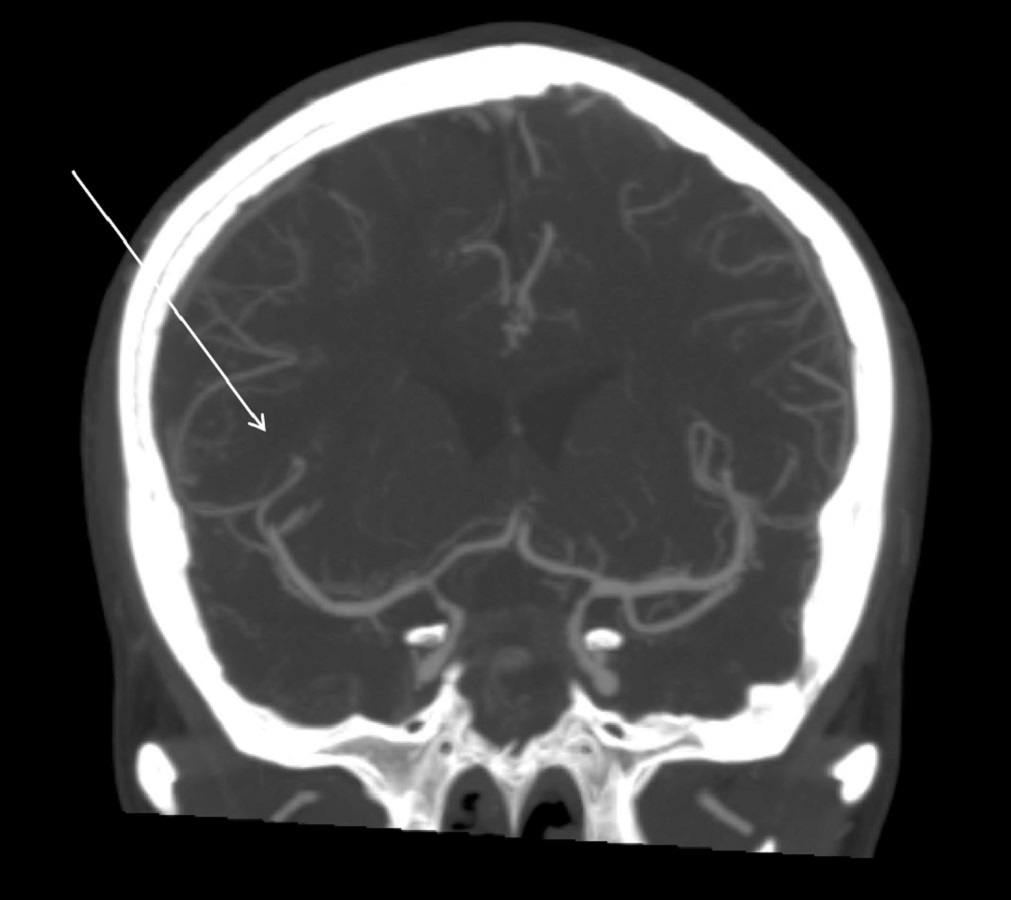
Computed tomography (CT) arteriography revealing significant vasospasm in the bilateral distal internal carotid arteries and distal basilar artery.

**Figure 5:**
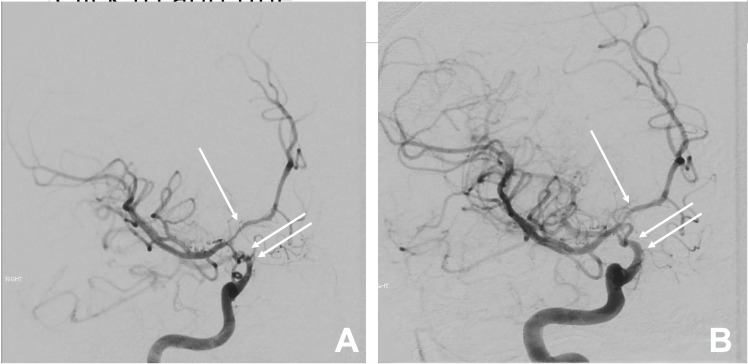
Angiogram demonstrating the distal right internal carotid artery pre-balloon angioplasty (A) and improved vascular caliber post-balloon angioplasty (B).

Given the patient’s acute bilateral vision loss, urgent endoscopic endonasal resection of the pituitary tumor was performed using standard image-guidance techniques. A cerebrospinal fluid (CSF) leak was noted postoperatively, which was repaired with Gelfoam, Durepair, and Surgicel. Histologic analysis revealed a pituitary adenoma with necrosis and hemorrhage (Figure 2). Postoperatively, he was given empiric prednisone 5 mg daily and levothyroxine 50 mg daily. He was given multiple doses of desmopressin (0.05 mg to 1 mg) to treat diabetes insipidus. His vision was unchanged from prior to operative intervention. The patient remained hospitalized due to the need for rehabilitation placement because of persistent visual deficits. On postoperative day seven, the patient was noted to have new onset left-sided weakness and left facial droop. Computed tomography (CT) revealed new acute infarcts involving the bilateral frontal lobes consistent with anterior cerebral artery territory (Figure 3). CT arteriography confirmed infarct in the distribution of the right anterior cerebral artery and detected significant vasospasm in the bilateral distal internal carotid arteries and distal basilar artery (Figure 4). The patient was treated with induced hypertension followed by operative intervention for definitive therapy. Emergent intra-arterial administration of 10 mg of verapamil and 150 micrograms (mcg) of nitroglycerin into each of the internal carotid arteries was performed. These therapies were administered for their vasodilatory effects and utility in treating vasospasm; however, internal carotid artery vasospasm was noted to be unchanged. Angioplasty of the distal right internal carotid artery and the right A1 segment of the anterior cerebral artery was performed using a Sterling monorail balloon (Boston Scientific, Massachusetts, USA) measuring 2.0 centimeters (cm) x 12 millimeters (mm). The balloon was first inflated to normal infusion capacity over a period of one minute, and after 30 seconds, the balloon was deflated. A post-balloon angioplasty angiogram was then performed demonstrating improved vascular caliber in both distal right internal carotid artery and right A1 anterior cerebral artery (Figure 5). Postoperatively, improvement in motor exam was noted. However, on post-angiography day one, interval decline in motor exam was noted, and repeat intra-arterial verapamil was performed. Verapamil was infused into the right internal carotid artery, left internal carotid artery, basilar artery, and right common carotid artery at doses of 15 mg, 10 mg, 10 mg, and 10 mg, respectively, in 1 mg aliquots per minute. Improvement of vasospasm was noted with improved vessel caliber. Following the procedure, improved motor strength was noted on exam with 5/5 strength throughout.

Postoperative course

**Figure 6:**
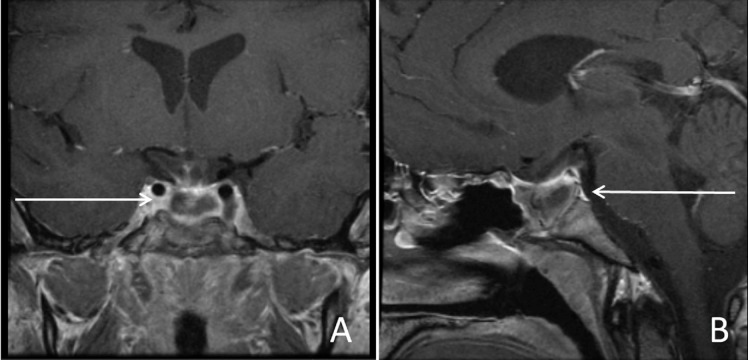
Postoperative magnetic resonance imaging (MRI) with contrast demonstrating minimal residual disease, coronal (A) and sagittal (B).

The patient’s postoperative course was marked by slow improvements in vision and near complete recovery of left-sided motor strength. At the five-month follow-up, continued improvement in vision was noted and he was free of any other neurological deficits. MRI showed minimal residual disease versus postoperative scarring in the sella (Figure 6). An ophthalmology follow-up at seven months revealed resolution of his left CN VI palsy, no light perception in the right eye, and improved vision of the left eye with visual acuity 20/25. He currently continues to receive treatment with prednisone 5 mg, levothyroxine 50 mcg, desmopressin 0.05 mg each morning and 0.1 mg each night, and intramuscular testosterone 200 mg every two weeks for panhypopituitarism. The patient follows up with endocrinology every six months and is expected to require long-term hormone replacement as well as continued surveillance imaging of the pituitary region annually.

## Discussion

Pituitary apoplexy followed by cerebral infarction is a rare occurrence. The mechanism of infarction may be attributed to vasospasm, mechanical compression of an intracranial vessel, or hypothalamic injury [3].

Vasospasm may be the result of subarachnoid hemorrhage or release of vasoactive substances from the tumor [4]. The vasoactive substances proposed to be released from the tumor include endothelin, angiotensin, and sphingosine metabolites [4-5]. In the instance of hypothalamic injury, the damaged hypothalamic tissue is also purported to release vasoactive compounds [4].

Vasospasm has also been reported as a consequence of pituitary tumor resection. Due to tumor proximity to the Circle of Willis and internal carotid arteries, vasospasm may be induced by surgical manipulation [2, 6]. In 2014, Chong et al. reported a review of the literature that reveals 22 cases of cerebral vasospasm following surgery for a pituitary tumor [5]. All instances occurred within 14 days postoperatively, with most cases presenting with hemiparesis [5]. In this case, the patient developed vasospasm while still in the hospital awaiting rehabilitation placement, which allowed for immediate diagnosis and treatment. The etiologies of vasospasm proposed include compression of the internal carotid artery, hemorrhagic enlargement of the pituitary gland, iatrogenic injury to vasculature, hypothalamic dysfunction, and release of vasoactive substances [5].

The treatment options for vasospasm following pituitary surgery include hemodynamic therapy, intra-arterial vasodilators, or angioplasty [5, 7]. Medical management via hemodynamic therapy focuses on euvolemic hypertension. However, in patients with diabetes insipidus, such therapy is challenging due to hypovolemia [7]. Although induction of a hypertensive state does not treat vasospasm, the cerebral blood flow is increased, helping to reduce ischemia. An intra-arterial vasodilator delivered by a catheter, most commonly verapamil, provides a short-lived effect on increasing blood vessel caliber, and therefore, improving blood flow to reduce ischemia [8]. Angioplasty, through dilation of blood vessels, restores cerebral blood flow and provides a more stable resolution of stenosis compared to intra-arterial medications, which often have short half-lives [8]. Success has been reported in the use of angioplasty to treat vasospasm following subarachnoid hemorrhage and transsphenoidal resection of pituitary tumors [9-10].

## Conclusions

We report a novel case of vasospasm that developed following transsphenoidal decompressive surgery for pituitary apoplexy. Our patient was treated with intra-arterial verapamil and angioplasty and experienced complete recovery of motor deficits. The authors emphasize the importance of recognizing that vasospasm can be a serious, delayed complication of apoplexy or pituitary surgery. This diagnosis should be considered and treated aggressively and with urgency once diagnosed. We propose a novel treatment for postoperative vasospasm following transphenoidal surgery for pituitary apoplexy, which was extremely successful in this reported case. The success achieved in the cases was made possible by the collaborative efforts of tumor surgeons, endovascular surgeons, critical care physicians, and endocrinologists.
